# Roundup Revelation: Weed Killer Adjuvants May Boost Toxicity

**Published:** 2005-06

**Authors:** Dorothy Bonn

Although the glyphosate-based herbicide Roundup is generally thought to be less toxic to the ecosystem than other pesticides, concerns about its effects on human reproduction persist. In a study in Ontario, Canada, exposure of male farmers to glyphosate-based herbicides was associated with an increase in miscarriage and premature birth in farm families. Seeking an explanation for these pregnancy-related problems, researchers at France’s Université de Caen investigated the effects of the full Roundup formulation and glyphosate alone on cultured human placental cells **[*EHP*** **113:716–720]**. The herbicide, they found, killed the cells at concentrations far below those used in agricultural practice. Surprisingly, they also found that Roundup was at least twice as toxic as glyphosate alone.

Virtually all previous testing of Roundup for long-term health damage has been done on glyphosate rather than on the full herbicide formulation, of which glyphosate makes up only around 40%. The remainder consists of inactive ingredients including adjuvants, chemicals that are added to improve the performance of the active ingredient. Roundup’s main adjuvant is the surfactant polyethoxylated tallowamine, which helps glyphosate penetrate plant cells.

The Roundup concentration recommended for agricultural use is 1–2% in water. The authors incubated placental cells with various concentrations of Roundup (up to 2.0%) or equivalent concentrations of glyphosate. The viability of the cells was measured after 18, 24, and 48 hours. No one is sure how Roundup interferes with reproduction, so the team also tested whether it, like other pesticides, would disrupt the activity of aromatase (an enzyme that regulates estrogen synthesis) in placental cells. Aromatase activity was measured after 1 hour and 18 hours.

The researchers found that a 2.0% concentration of Roundup and an equivalent concentration of glyphosate killed 90% of the cultured cells after 18 hours’ incubation. The median lethal dose for Roundup (0.7%) was nearly half that for glyphosate, meaning Roundup was nearly twice as toxic as the single chemical alone. Further, the viability of cells exposed to glyphosate was considerably reduced when even minute dilutions of Roundup were added.

After an hour’s incubation with Roundup, estrogen synthesis in placental cells (as shown by aromatase activity) was enhanced by about 40%. After 18 hours, however, synthesis was inhibited, perhaps reflecting an effect on aromatase gene expression. This effect was not seen with glyphosate alone.

The study showed that the effect of Roundup on cell viability increased with time and was obtained with concentrations of the formulation 10 times lower than those recommended for agricultural use. Roundup also disrupted aromatase activity at concentrations 100 times lower than those used in agriculture. The researchers suspect that the adjuvants used in Roundup enhance the bioavailability and/or bioaccumulation of glyphosate.

How these findings translate into activity of Roundup in the human body is hard to say. The French researchers point out that serum proteins can bind to chemicals and reduce their availability—and therefore their toxicity—to cells. Nevertheless, the authors conclude that the demonstrated toxicity of Roundup, even at concentrations below those in agricultural use, could contribute to some reproduction problems.

